# Trends of Vancomycin-Resistant Enterococcus Infections in Cancer Patients

**DOI:** 10.7759/cureus.31335

**Published:** 2022-11-10

**Authors:** Abdur Rafey, Summiya Nizamuddin, Waleed Qureshi, Ali Anjum, Azra Parveen

**Affiliations:** 1 Department of Internal Medicine and Infectious Diseases, Shaukat Khanum Memorial Cancer Hospital and Research Center, Lahore, PAK; 2 Department of Pathology, Shaukat Khanum Memorial Cancer Hospital and Research Center, Lahore, PAK; 3 Department of Internal Medicine, Shaukat Khanum Memorial Cancer Hospital and Research Center, Lahore, PAK

**Keywords:** febrile neutropenia, malignancy, linezolid, enterococcus, vancomycin

## Abstract

Objective

Vancomycin-resistant *Enterococcus* (VRE) is an important cause of infection in immunocompromised populations. In Pakistan, very limited data are available regarding *Enterococcus* infection and its outcomes. We conducted this study to evaluate the trends including risk factors, treatment options, and outcomes of infections due to vancomycin-resistant enterococci in cancer patients in Pakistan.

Methods

We conducted a retrospective observational study. We extracted data from medical records of our center over a period of seven years. All admitted cancer patients with any vancomycin-resistant *Enterococcus *positive culture were included. The following parameters were evaluated: age, gender, type of cancer, febrile neutropenia, prior antibiotics, admission, comorbidities, system-wise infections (including bacteremia, catheter-related infection, pneumonia, urinary tract infections, intra-abdominal infection, bone and joint infections, skin and skin structure infections), intensive care unit admission, and 30-day all-cause mortality. Frequencies of infections, mortality, and drug susceptibility were evaluated over the course of seven years.

Results

Risk factors for *enterococcal* infection included prior exposure of piperacillin/tazobactam (n=209, 86.7%), meropenem (n=132, 54.8%), vancomycin (n=126, 52.3%), metronidazole (n=67, 27.8%), prior admission for more than 48 hours (n=198, 82.2%), and comorbidities (n=76, 31.5%), with acute kidney injury being most common (n=72, 95%) followed by diabetes mellitus (n=70, 92.1%). Precursor B cell acute lymphoblastic leukemia (pre-B ALL) was the most common malignancy in which infection occurred (n=54, 38.3%). Among patients who developed infection, 46% (n=111) had febrile neutropenia. *Enterococcus species* caused infection in 61% (n=147) and *Enterococcus faecium* in 39% (n=94). Bacteremia occurred in 45.2% (n=109) patients followed by urinary tract and intra-abdominal infection; 45.6% (n=110) patients were admitted to ICU, and 30-day all-cause mortality was 44.8% (n=108). Linezolid sensitivity was 100%. The total number of enterococci infections decreased over seven years. Frequency of *E. species* infection, bacteremia, intra-abdominal, skin-related infections, and recurrent infection also decreased, but the frequency of *E. facium* infections, ICU admission, and 30-day all-cause mortality was increased.

Conclusion

VRE infections have become less frequent but more severe in recent years with increase in mortality. Prior use of antibiotics (including piperacillin/tazobactam, vancomycin, carbapenems, and metronidazole), diagnosis of hematological malignancy, febrile neutropenia, diabetes mellitus, and renal failure are the risk factors for VRE infection. Bacteremia was the most common infection with high mortality rate. All strains remain sensitive to linezolid. Patients with these risk factors should be worked up for VRE and can be treated with linezolid empirically.

## Introduction

*Enterococci* are gram-positive facultative anaerobic cocci that exist in short and medium chains [1]. They are usually found in the gastrointestinal tract and tend to cause serious disease in the immunocompromised or those with underlying diseases. The most common infections involve the urinary tract, wounds, and the bloodstream. *Enterococcus faecalis* (*E. faecalis*) is the most common species isolated, followed by *Enterococcus faecium* (*E. faecium*) [2]. All enterococci are intrinsically resistant to penicillins, cephalosporins, and carbapenems [3].

Vancomycin-resistant *enterococci* (VRE) are of six types: Van A, Van B, Van C, Van D, Van E, and Van G. Van A resistance phenotype has high-level vancomycin/teicoplanin resistance, and van B resistance phenotype has variable levels of vancomycin resistance but is susceptible to teicoplanin [4].

One of the risk factors of VRE colonization and infection are prior use of broad-spectrum antibiotics including vancomycin, ceftazidime [5-7], ciprofloxacin [6-8], meropenem [9], and metronidazole [10-13]. Other risk factors include previous hospitalization, chronic heart failure, chronic obstructive pulmonary disease, chronic renal failure, malignancy [5], diabetes mellitus, previous enterococcal infection (both vancomycin sensitive or resistant), VRE-positive patients in nearby beds, diarrhea [7,9,11,14], *Clostridium difficile* infection [14,15], invasive catheter [12], and previous chemotherapy [13]. A study showed that *E. faecium* (van A type) was isolated from all hematological cancer and febrile neutropenia patients, with bacteremia being the most common infection [16]. Patients with acute myelogenous leukemia, transplant recipients, or severely ill patients also have an increased risk of developing infection [17,18].

*Enterococci* caused bloodstream infections in 33.8% of transplant patients [19] and 29.2% of cancer patients [20], intra-abdominal infections in 10.5% [21], and urinary tract and skin structure infection in less 10% of patients [22].

Mortality rate for patients with VRE infection remains high, as mortality rates of 24.5% [22], 35.7% [23], 36% [24], and 57.1% [16] have been documented in different studies.

Quinupristin/dalfopristin and linezolid have emerged as approved therapeutic options for VRE [24,25,17], but quinupristin-dalfopristin has limited activity against VRE due to agricultural use of streptogramin and there is emerging linezolid resistance. Nitrofurantoin and fosfomycin are alternatives in uncomplicated VRE urinary tract infection. Daptomycin and tigecycline have shown excellent potential for treating VRE infection. Other treatment options include chloramphenicol, doxycycline, high-dose ampicillin or ampicillin/sulbactam, and nitrofurantoin (for lower urinary tract infection) [25].

We conducted a retrospective observational study to evaluate the risk factors and outcomes of VRE infections in cancer patients.

## Materials and methods

We conducted a retrospective single-center study at Shaukat Khanum Memorial Cancer Hospital and Research Center (SKMCH&RC), a tertiary care cancer center in Lahore, Pakistan. The study was approved by the Institutional Review Board at SKMCH&RC with a waiver of informed consent.

All registered cancer patients with a positive culture for VRE, from January 1, 2015, to December 31, 2021, were included in the study. Data were extracted from the online medical records. Patients were evaluated for fever and neutropenia. We also evaluated blood, urine, sputum or bronchoalveolar lavage, and other site-specific pus or tissue cultures. Computed tomography (CT) scan of the abdomen and pelvis with contrast was performed for suspected intra-abdominal infections, with radiological guided pus aspiration if applicable. Magnetic resonance imaging (MRI) with contrast of the involved bone and joint was performed followed by radiological aspiration of fluid collections for involved joints.

All febrile patients were empirically treated with piperacillin-tazobactam. Patients with hemodynamic instability were started on empirical carbapenems. Empirical vancomycin was also added for patients with hemodynamic instability, chest X-ray consolidation, neurological symptoms, evidence of skin infection, and any central venous catheter in place. Cultures were followed for identification of organisms and sensitivity. Data on the following variables were included: age, gender, hematological or solid organ cancer, febrile neutropenia, antibiotics given within the last three months for more than 48 hours, details of isolates from positive cultures and their sensitivities, prior admission lasting more than seven days in last two months, any comorbidities, and any prior VRE infection. System-wise infections including bacteremia, catheter-related infection, pneumonia, urinary tract infections (including catheter- or stent-related), intra-abdominal infection, bone and joint infections, skin and skin structure infections, intensive care unit admission, and 30-day all-cause mortality were also evaluated. Frequencies and proportions were reported for categorical variables. Mean and standard deviation were reported for continuous variables.

## Results

A total of 241 patients were included in this study. The mean age was 32 years with a standard deviation of 23. There were 144 (59.75%) male patients and 97 (40.2%) female patients. Adult patients (aged more than 18 years) were 154 (63.9%) and children were 87 (36.1%). Hematological malignancies were diagnosed in 141 (58.5%) and solid organ malignancies were diagnosed in 100 (41.5%) patients. The most common hematological malignancy was precursor B cell acute lymphoblastic leukemia (pre-B ALL), which was present in 54 (38.3%), and the most common solid organ malignancies were breast cancer, urinary bladder cancer, and osteosarcoma, which were present in 11 (11%) patients each. A total of 111 (46%) patients developed febrile neutropenia after chemotherapy. Results are summarized in Table 1.

**Table 1:** Baseline patient demographics pre-B ALL, precursor B cell acute lymphoblastic leukemia; DLBCL, diffuse large B cell lymphoma; AML, acute myeloid leukemia; CLL, chronic lymphocytic leukemia; CA, cancer

Demographic characteristics	N (%), N=241
Male	144 (59.75%)
Female	97 (40.2%)
Adults	154 (63.9%)
Children	87 (36.1%)
Hematological cancer	141 (58.5%)
Pre-B ALL	54 (38.3%)
Burkitt’s lymphoma	35 (24.8%)
DLBCL	19 (13.5%)
Hodgkin lymphoma	12 (8.5%)
AML	9 (6.4%)
CLL	8 (5.7%)
Multiple myeloma	4 (2.8%)
Solid organ caner	100 (41.5%)
CA breast	11 (11%)
CA urinary bladder	11 (11%)
Osteosarcoma	11 (11%)
CA colon	10 (10%)
Renal cell CA	10 (10%)
CA ovary	10 (10%)
CA esophagus	8 (8%)
CA pancreas	7 (7%)
CA lung	7 (7%)
CA prostate	6 (6%)
CA stomach	5 (5%)
CA gallbladder	4 (4%)
Neutropenia	111 (46%)

We found that 86.7% (n=209) patients received prior piperacillin/tazobactam, 54.8% (n=132) received meropenem, 52.3% (n=126) received vancomycin, 27.8% (n=67) received metronidazole, 27% (n=65) received ciprofloxacin, 23.7% (n=57) received imipenem/cilastatin, 22% (n=53) received ceftriaxone, and 17.4% (n=42) received teicoplanin for more than 48 hours’ duration within the last three months. A total of 198 (82.2%) patients had prior admission for more than seven days in the last two months; 31.5% (n=76) of patients had comorbidities, with the most common being acute kidney injury (n=72, 95%) due to any cause, followed by diabetes mellitus (n=70, 92.1%). Prior VRE infections occurred in 10.8% (n=26) patients. Results are summarized in Table 2.

**Table 2:** Prior antibiotics and other risk factors for VRE infection AKI, acute kidney injury; DM, diabetes mellitus; IHD, ischemic heart disease; VRE, vancomycin-resistant *Enterococcus*

Risk factors	N (%), N=241
Prior antibiotic exposure for more than 48 hours	
Piperacillin/tazobactam	209 (86.70%)
Meropenem	132 (54.80%)
Vancomycin	126 (52.30%)
Metronidazole	67 (27.80%)
Ciprofloxacin	65 (27%)
Imipenem	57 (23.70%)
Ceftriaxone	53 (22%)
Teicoplanin	42 (17.40%)
Colistimethate sodium	35 (14.50%)
Azithromycin	30 (12.40%)
Amikacin	20 (8.30%)
Clarithromycin/Co-amoxiclav	9 (3.70%)
Fosfomycin	8 (3.30%)
Linezolid	2 (0.80%)
Comorbidities	76 (31.5%)
AKI	72 (95%)
DM	70 (92.1%)
AKI+IHD	7 (9.2%)
Clostridium difficile	3 (1.24%)
IHD	1 (1.3%)
Prior admission for > than 7 days in 2 months	198 (82.2%)
Recurrence	26 (10.8%)

Out of all isolates, 147 (61%) were *Enterococcus species* (*E. species*) and 94 (39%) were *E. faecium*. Bacteremia occurred in 109 (45.2%) patients, out of which 61 (56%) were associated with central catheter. Urinary tract infections occurred in 78 (32.4%) patients, out of which 33 (42.3%) were not related to catheter or urological procedure. Intra-abdominal infection occurred in 18 (7.5%) patients, out of which 10 (56%) occurred after surgery, six (33.3%) were associated with intra-abdominal abscesses, and two (11%) were associated with peritonitis. Pneumonia occurred in 16 (6.6%) patients. Skin and skin structure-related infection occurred in 15 (6.2%) patients, out of which inguinal and sacral cellulitis occurred in 13 (86.7%) patients, and laparotomy-associated surgical site infection occurred in two (13.3%) patients. Sinusitis occurred in eight (3.3%) patients in the presence of febrile neutropenia. A total of 110 (45.6%) patients were admitted to ICU, and 30-day all-cause mortality was 44.8% (n=108). Results are summarized in Table 3.

**Table 3:** Incidence of VRE and site-wise infection, ICU admission, and 30-day all-cause mortality PCN, percutaneous nephrostomy; SSI, surgical site infection; ICU, intensive care unit; VRE, vancomycin-resistant Enterococcus

Infection characteristics	N (%), N=241
Enterococcus species	147 (61%)
Enterococcus faecium	94 (39%)
Bacteremia	Total: 109 (45.2%)
	Central line-related: 61 (56%)
	Spontaneous: 48 (44%)
Urinary tract infection	Total: 78 (32.40%)
	Spontaneous: 33 (42.3%)
	Catheter-related: 31 (39.7%)
	PCN-related: 8 (10.3%)
	Stent-related: 6 (7.7%)
Intra-abdominal infections	Total: 18 (7.5%)
	Post-surgery: 10 (56%)
	Abscess-related: 6 (33.3%)
	Ascites-related: 2 (11%)
Pneumonia	16 (6.60%)
Skin and skin-related infections	Total: 15 (6.2%)
	Inguinal and sacral cellulitis: 13 (86.7%)
	Laparotomy/SSI-related: 2 (13.3%)
Sinusitis	8 (3.3%)
Bone and joint	Discitis: 1 (0.4%)
ICU admission	110 (45.60%)
30-day mortality	108 (44.80%)

As far as sensitivities were concerned, 100% (n=241) VRE were sensitive to linezolid, 64.7% (n=156) were sensitive to chloramphenicol, and 18.3% (n=44) were sensitive to tetracycline. Results are summarized in Table 4.

**Table 4:** VRE sensitivities VRE, vancomycin-resistant *Enterococcus*

Drug	Sensitivity
Linezolid	241 (100%)
Chloramphenicol	156 (64.70%)
Tetracycline	44 (18.30%)
Fosfomycin	39 (16.20%)
Nitrofurantoin	27 (11.20%)
Tigecycline	25 (10.40%)

Over a period of seven years, the total number of patients diagnosed with VRE infection decreased from 50 cases in 2015 to 26 cases in 2021. Frequency of E. species infection decreased from 66% to 62%, but the frequency of E. faecium infections increased from 34% to 38%. Frequency of bacteremia decreased from 54% to 31%, and that of intra-abdominal infections decreased from 4% to 2%. Frequency of urinary tract infections increased from 32% to 35%. Frequency of ICU admission increased from 26% to 35%, and that of mortality increased from 24% to 35%. Results are summarized in Table 5.

**Table 5:** Trends of VRE infection UTI, urinary tract infection; IAI, intra-abdominal infection; SSRI, skin and skin-related infection; AKI, acute kidney injury; DM, diabetes mellitus; IHD, ischemic heart disease; pre-B ALL, precursor B cell acute lymphoblastic leukemia; DLBCL, diffuse large B cell lymphoma; AML, acute myeloid leukemia; CLL, chronic lymphocytic leukemia; CA, cancer; VRE, vancomycin-resistant *Enterococcus*

	2015	2016	2017	2018	2019	2020	2021
	N=50	N=31	N=51	N=36	N=29	N=18	N=26
	N (%)	N (%)	N (%)	N (%)	N (%)	N (%)	N (%)
Infection characteristics							
E. species	33 (66%)	29 (93.5%)	31 (61%)	14 (39%)	17 (58.6%)	7 (39%)	16 (62%)
E. faecium	17 (34%)	2 (6.5%)	20 (39%)	22 (61%)	12 (41.4%)	11 (61%)	10 (38%)
Bacteremia	27 (54%)	16 (52%)	14 (27%)	19 (52%)	15 (52%)	10 (56%)	8 (31%)
UTI	16 (32%)	12 (39%)	22 (43%)	5 (14%)	6 (21%)	8 (44%)	9 (35%)
IAI	4 (8%)	2 (6.5%)	3 (6%)	6 (17%)	1 (3.4%)	-	2 (7.7%)
Pneumonia	2 (4%)	4 (13%)	1 (2%)	3 (8.3%)	3 (10%)	-	3 (11.5%)
SSRI	4 (8%)	1 (3.2%)	5 (10%)	2 (5.6%)	-	-	3 (11.5%)
Bone and joint infection	-	-	-	1 (2.7%)	-	-	-
ICU admission	13 (26%)	18 (58%)	25 (49%)	23 (64%)	14 (48%)	8 (44%)	9 (35%)
30-day mortality	12 (24%)	18 (58%)	25 (49%)	23 (64%)	13( 45%)	8 (44%)	9 (35%)
Comorbidities							
AKI	7 (14%)	11 (35%)	14 (27%)	16 (44%)	9 (31%)	5 (28%)	10 (38%)
DM	7 (14%)	10 (32%)	14 (27%)	15 (42%)	9 (31%)	5 (28%)	10 (38%)
AKI+IHD	4 (8%)	-	1 (2%)	-	1 (3.4%)	1 (5.6%)	-
Clostridium difficile	1 (2%)	-	-	-	2 (7%)	-	-
IHD	-	-	-	1 (2.7%)	-	-	-
Prior admission	41 (82%)	24 (77%)	40 (78%)	32 (89%)	27 (93%)	13 (72%)	21 (81%)
Recurrence	8 (16%)	3 (9.7%)	8 (16%)	2 (5.6%)	1 (3.4%)	1 (5.6%)	3 (11.5%)
Malignancy							
Pre-B ALL	13 (26%)	6 (19%)	14 (27%)	11 (30.1%)	1 (3.4%)	3 (16.7%)	6 (23%)
Burkitt’s lymphoma	10 (20%)	4 (13%)	5 (10%)	5 (13.9%)	4 (14%)	6 (33.3%)	1 (3.8%)
DLBCL	7 (14%)	8 (26%)	-	-	4 (14%)	-	-
Hodgkin lymphoma	3 (6%)	5 (16%)	2 (4%)	-	-	-	2 (7.7%)
AML	1 (2%)	1 (3.2%)	2 (4%)	-	-	-	5 (19%)
CLL	2 (4%)	-	-	3 (8.3%)	2 (7%)	1 (5.6%)	-
Multiple myeloma	1 (2%)	-	-	3 (8.3%)	-	-	-
CA breast	2 (4%)	-	2 (4%)	2 (5.6%)	1 (3.4%)	2 (11%)	2 (7.7%)
CA urinary bladder	2 (4%)	-	4 (8%)	2 (5.6%)	1 (3.4%)	2 (11%)	-
Osteosarcoma	2 (4%)	3 (9.7%)	1 (2%)	1 (2.7%)	2 (7%)	2 (11%)	-
CA colon	2 (4%)	1 (3.2%)	-	-	4 (14%)	2 (11%)	1 (3.8%)
Renal cell CA	3 (6%)	-	4 (8%)	2 (5.6%)	-	-	1 (3.8%)
CA ovary	1 (2%)	3 (9.7%)	2 (4%)	-	1 (3.4%)	1 (5.6%)	2 (7.7%)
CA esophagus	-	2 (6.5%)	1 (2%)	1 (2.7%)	1 (3.4%)	2 (11%)	1 (3.8%)
CA pancreas	2 (4%)	-	-	4 (11%)	-	1 (5.6%)	
CA lung	-	2 (6.5%)	2 (4%)	1 (2.7%)	1 (3.4%)	1 (5.6%)	-
CA prostate	-	-	3 (6%)	2 (5.6%)	1 (3.4%)	-	-
CA stomach	-	-	2 (4%)	2 (5.6%)	1 (3.4%)	-	-
CA gallbladder	-	1 (3.2%)	-	1 (2.7%)	-	1 (5.6%)	1 (3.8%)

Frequency of fosfomycin and chloramphenicol sensitivity decreased from 20% to 15% and 62% to 54%, respectively, whereas sensitivity of tetracycline and nitrofurantoin increased from 8% to 31% and 12% to 19%, respectively. Results are summarized in Table 6.

**Table 6:** Trends of VRE drug sensitivity VRE, vancomycin-resistant *Enterococcus*

	2015	2016	2017	2018	2019	2020	2021
	Sensitivity						
	N=50	N=31	N=51	N=36	N=29	N=18	N=26
	N (%)	N (%)	N (%)	N (%)	N (%)	N (%)	N (%)
Drug							
Linezolid	50 (100%)	31 (100%)	51 (100%)	36 (100%)	29 (100%)	18 (100%)	26 (100%)
Chloramphenicol	31 (62%)	25 (81%)	26 (51%)	31 (86%)	19 (65%)	10 (56%)	14 (54%)
Tetracycline	4 (8%)	1 (3.2%)	5 (9.8%)	12 (33%)	9 (31%)	5 (28%)	8 (31%)
Fosfomycin	10 (20%)	1 (3.2%)	11 (22%)	1 (3%)	6 (21%)	6 (33%)	4 (15%)
Nitrofurantoin	6 (12%)	1 (3.2%)	13 (25%)	-	-	2 (11%)	5 (19%)
Tigecycline	-	11 (35%)	14 (27%)	-	-	-	-

## Discussion

The results of our study revealed that the total number of patients with VRE infections has decreased but related ICU admission and mortality had increased in recent years. Most common infections were caused by *E. species* (n=147, 61%). VRE mainly caused infections in adults, and pre-B ALL was the most common malignancy. Other risk factors for VRE infection were febrile neutropenia, prior use of antibiotics, prior admission for more than seven days, and acute kidney injury. Bacteremia was the most common infection followed by UTI and intra-abdominal infections. In neutropenic patients, sinusitis was also reported (n=8, 3.3%). VRE infection led to 45.6% patients being admitted to ICU, with high 30-day all-cause mortality of 44.8% (n=108). All of the VRE isolates were sensitive to linezolid followed by chloramphenicol and tetracycline.

Our results confirmed the findings of prior studies that the use of broad-spectrum antibiotics [5-7,9-13], previous hospitalization, ischemic heart disease, renal failure, hematological malignancy, diabetes mellitus [7,9, 11,14], and previous chemotherapy [13] increased the risk of VRE.

Similar to the results of prior studies, our study also concluded that bacteremia was the most common infection [19,20,25] followed by UTI and intra-abdominal infections [21,22] with high mortality rates of up to 57.1% [16]. All VRE isolates were sensitive to linezolid, thus making linezolid the drug of choice just like other studies [24,25,17].

There were some contradicting results as well. We did not find chronic obstructive pulmonary disease [7,9,11,14] as a risk factor for VRE. In our results, previous enterococcal infection occurred in only 10.8% (n=26) patients, whereas diarrhea [7,9,11,14] and Clostridium difficile infection [15] occurred in 1.24% (n=3) patients only. There were no patients with endocarditis [26]. Although nitrofurantoin and fosfomycin are alternatives in uncomplicated VRE urinary tract infections, their sensitivities were only 11.20% and 16.20%, respectively. Tigecycline has been reported to show excellent potential for treating VRE infection [25]; however, in our study, only 10.40% (n=25) VRE were susceptible to tigycycline, whereas 18.30% (n=44) were sensitive to tetracycline; 64.70% (n=156) isolates were sensitive to chloramphenicol [25], but its use is limited due to its toxicity. Being a resource-limited country, we do not have access to quinupristin/dalfopristin and daptomycin as treatment options for VRE. VRE without resistance to aminoglycosides can be treated with high-dose ampicillin combined with an aminoglycoside [27], making it a treatment option but requires further studies.

Major weaknesses of our study are its single-center nature and retrospective study design. We recommend further multicenter meta-analysis, retrospective case-control studies, and prospective studies for further VRE trend evaluation. The strengths of our study are the sample size and the study period spanning over seven years, in a region with minimal prior literature available on trends of VRE.

## Conclusions

VRE infections have become less frequent but more severe with increasing mortality. Risk factors for VRE infection include prior use of piperacillin/tazobactam, vancomycin, carbapenems, and metronidazole, diagnosis of hematological malignancy, febrile neutropenia, diabetes mellitus, and renal failure. VRE bacteremia was the most common infection with high mortality rate of 44.8%; therefore, if patients with these risk factors develop fever, there should be a high suspicion of VRE infection, and they should undergo early workup. All VRE strains were sensitive to linezolid, thus making it an empirical drug of choice in life-threatening conditions. However, further multicenter prospective studies are warranted to make treatment guidelines and further trends evaluation.
